# Exopolysaccharides as Antimicrobial Agents: Mechanism and Spectrum of Activity

**DOI:** 10.3389/fmicb.2021.664395

**Published:** 2021-05-19

**Authors:** Abdelmoneim K. Abdalla, Mutamed M. Ayyash, Amin N. Olaimat, Tareq M. Osaili, Anas A. Al-Nabulsi, Nagendra P. Shah, Richard Holley

**Affiliations:** ^1^Food Science Department, College of Agriculture, South Valley University, Qena, Egypt; ^2^Department of Food Science, College of Food and Agriculture, United Arab Emirates University (UAEU), Al Ain, United Arab Emirates; ^3^Department of Clinical Nutrition and Dietetics, Faculty of Applied Medical Sciences, The Hashemite University, Zarqa, Jordan; ^4^Department Clinical Nutrition and Dietetics, University of Sharjah, Sharjah, United Arab Emirates; ^5^Department of Nutrition and Food Technology, Jordan University of Science and Technology, Irbid, Jordan; ^6^Food and Nutritional Science, School of Biological Sciences, The University of Hong Kong, Hong Kong, Hong Kong; ^7^Department of Food and Human Nutritional Sciences, University of Manitoba, Winnipeg, MB, Canada

**Keywords:** antibacterial, antiviral, antifungal, biopolymers, biopreservative

## Abstract

Exopolysaccharides (EPSs) are metabolites synthesized and excreted by a variety of microorganisms, including lactic acid bacteria (LAB). EPS serve several biological functions such as interactions between bacteria and their environments, protection against hostile conditions including dehydration, the alleviation of the action of toxic compounds (bile salts, hydrolyzing enzymes, lysozyme, gastric, and pancreatic enzymes, metal ions, antibiotics), and stresses (changing pH, osmolarity), and evasion of the immune response and phage attack. Bacterial EPSs are considered valuable by the food, pharmaceutical, and nutraceutical industries, owing to their health-promoting benefits and rheological impacts. Numerous studies have reported the unusual antimicrobial activities of various EPS against a wide variety of pathogenic microbes (bacteria, virus, and fungi). This review aims to provide a comprehensive examination of the *in vitro* and *in vivo* antimicrobial activities of different EPSs, mainly against foodborne bacterial, fungal, and viral pathogens. The mechanism of EPS action against these pathogens as well as the methods used to measure antimicrobial activities are critically reviewed.

## Introduction

Polysaccharides or glycans are abundant in nature and exhibit varied chemical structures, physical properties and biological functions. They can be obtained from plants, animals, algae, and microorganisms, including fungi and bacteria (Moscovici, 2015). Most bacterial cells are covered by a layer of polysaccharides known as the *glycocalyx*. When these polymers are attached to the cell surface through covalent bonds to create a capsule, they are called capsular polysaccharides (CPS). Others are loosely attached to the cell surface, or they are entirely released to the surroundings, creating slime, which is also referred to as exopolysaccharide (EPS) (Hidalgo-Cantabrana et al., 2012; Lynch et al., 2018). EPSs are classified into homo-EPS (HoEPS) composed of one repeated monosaccharide unit and hetero-EPS (HeEPS) composed of more than one monosaccharides unit (Zhou et al., 2019). It was suggested that EPS serves several purposes, including enhanced interactions between bacteria and their environment, protection against the development of hostile conditions, the effects of toxic compounds (bile salts, hydrolyzing enzymes, lysozyme, gastric and pancreatic enzymes, metal ions, antibiotics), and environmental stresses (changes in pH, temperature or osmolarity), evasion of the immune response in animals and phage attack (Castro-Bravo et al., 2017; Lynch et al., 2018). Nonetheless, in microbial ecology, their real functions are still unknown.

Lactic acid bacteria (LAB) represent a part of the human microbiota of mucous membranes such as the gastrointestinal and urinary tracts. They have potential positive influences on these ecosystems through stimulating the immune system and enhancing human resistance against infections of viral and bacterial pathogens. The LAB-derived EPSs are considered natural compounds with various biological activities, including antioxidants, anticancer, antidiabetic, immunomudolatory effects, and antimicrobials (Al Kassaa et al., 2014; Riaz Rajoka et al., 2020). LABs are known to be a source of numerous biologically active substances that differ in chemical structure, including carbon dioxide, organic acids, bacteriocins, and wide range of molecular weight compounds, for instance, antifungal peptides, reuterin, reutericyclin as well as EPS (Li et al., 2014).

Lactic acid bacteria-derived EPSs are generally regarded favorably by the food, pharmaceutical, and nutraceutical industries owing to their rheological impacts and health-promoting properties (Moscovici, 2015; Caggianiello et al., 2016; Lynch et al., 2018; Riaz Rajoka et al., 2020). Recently, significant numbers of LAB-derived EPSs have been reported, and their composition, structure, biosynthesis, and functional properties have been examined in detail (Saadat et al., 2019; Zhou et al., 2019; Riaz Rajoka et al., 2020). As for their positive health attributes, EPSs have gained considerable attention recently. EPSs produced by LAB, including *Lactobacillus* spp., have potential functional properties that are important to the host’s health. They have been reported to possess several bio-functional effects, including the ability to scavenge a broad spectrum of free radicals, ability to bind free cholesterol. They can modulate gut microbiota, cause immunomodulation, and can have antitumor, antimicrobial, antibiofilm, and antitoxic effects, which may promote them as potential therapeutics (Nataraj et al., 2020; Riaz Rajoka et al., 2020; Sundararaman et al., 2020). However, the previous review papers about EPS focused mainly on the EPS structure, molecular weight, rheological properties, antioxidant, and monosaccharides composition. The assessment methods of the antimicrobial activity of the EPS were not criticized. Unlike the previous reviews, this review attempts to provide a comprehensive and in-depth examination of the *in vitro* and *in vivo* antimicrobial activities of LAB-derived EPS. The potential mechanism(s) of their antimicrobial activity is also reviewed. Finally, the methods used for the measurement of the antimicrobial activity of EPS are critically examined.

## Antimicrobial Activities of Native Lab-Derived EPS

### Antibacterial Activity and Mechanism of Action

Recently, LAB-derived EPS showed remarkable antimicrobial activity against bacterial pathogens, both Gram-positive and Gram-negative. However, the underlying functional mechanism requires more clarification. EPSs that can exert antagonistic activity against bacterial pathogens, as shown by numerous studies, are presented in Table 1. For example, under *in vitro* conditions, the EPS produced by *L. rhamnosus* (isolated from human breast milk) displayed substantial antibacterial activity against the pathogens *Salmonella enterica* serovar Typhimurium and *Escherichia coli* (Riaz Rajoka et al., 2018). EPS produced by *Bifidobacterium longum* was shown to impair the cell division of bacterial pathogens, namely *Vibrio parahaemolyticus*, *S.* Typhimurium, *Staphylococcus aureus*, and *Bacillus cereus* (Wu et al., 2010). In another work, *Lactobacillus gasseri* hetero-EPS (HeEPS) exhibited antibacterial activity against several pathogens, including *Listeria monocytogenes* MTCC 657, which was significantly inhibited (Rani et al., 2018). EPS-C70 produced by *L. plantarum* C70 isolated from camel milk caused a 2–3 log decline in *E. coli* and *S. aureus* viability when tested against these bacterial pathogens (Ayyash et al., 2020b).

**TABLE 1:** Antibacterial activity of LAB-derived EPS against Gram^+^ and Gram^–^ bacterial pathogens.

Genus	Specie/Strain	Monosaccharides	Mw (kDa)	Conc. mg/mL	Species targeted	Growth medium	Test Temp (°C)	Incubation time (h)	Da (mm)	MICb (mg/Ml)	Log RDc	References
**Against Gram-positive**												
*Lactobacillus*	*L. gasseri* FR4	Glu, Man, Gal, Rha, Fuc	186.0	10	*E. faecalis*	BHI agar	37	24	0.93	–	–	Rani et al., 2018
				10C	*E. faecalis*	BHI agar	37	24	1.49	–	–	
					*L. monocytogenes*	BHI agar	37	24	7.43	–	–	
					*L. monocytogenes*	BHI agar	37	24	9.14	–	–	
					*S. aureus*	BHI agar	37	24	3.16	–	–	
					*S. aureus*	BHI agar	37	24	5.86	–	–	
	*L. plantarum*	–	36.0	0.064 –1	*L. monocytogenes*	MHB broth	37	22–24	–	10	–	Mahdhi et al., 2017
				0.064 –1	*S. aureus*	MHB broth	37	22–24	–	2	–	
	*L. rhamnosus* SHA111, 113,114,115,116,117	–	–	5	*S. petrasii* subsp. *pragensis*	LB agar	37	48	9.3–13.6	–	–	Riaz Rajoka et al., 2018
	*L. vaginalis* SHA111	–	–	5	*S. petrasii* subsp. *pragensis*	LB agar	37	–	9.5	–	–	Riaz Rajoka et al., 2020
	*L. brevis*	–	–	0.05-0.01 M	*S. aureus*	N agar/N broth	28	48	9.53	0.0125		
	*L. plantarum* C70	Ara, Man, Glu, Gal	380.0	5	*L. monocytogenes*	BHI	37	18	–	–	2.84	Ayyash et al., 2020a
				5	*S. aureus*	BHI	37	18	–	–	3.61	
*Lactococcus*	*Lc. lactis* F-mou	–	–	–	*B. cereus*	MHB broth	30	–	–	6.25	–	Nehal et al., 2019
	*Lc. garvieae* C47	Glu, Ara, Xyl	7300.0	5	*L. monocytogenes*	N broth	37	24	–	–	2.84	Ayyash et al., 2020b
	*Lc. lactis* F-mou	–	–	–	*L. monocytogenes*	MHB broth	30	–	–	9.3	–	Nehal et al., 2019
	*Lc. garvieae* C47	Glu, Ara, Xyl	7300.0	5	*S. aureus*	N broth	37	24	–	–	3.05	Ayyash et al., 2020b
	*Lc. lactis* F-mou	–	–	–	*S. aureus*	MHB broth	37	–	–	8.5	–	Nehal et al., 2019
*Streptococcus*	*S. thermophilus* GST-6	Gluc and gal	–	10S	*S. aureus*	BHI agar	37	24	17.8	2	–	Zhang et al., 2016
				10	*S. aureus*	BHI agar	37	24	10.2	6	–	
**Against Gram-Negative**												
*Lactobacillus*	*L. gasseri* FR4	Glu, Man, Gal, Rha, Fuc	186.0	10	*E. coli*	BHI agar	37	24	1.49	–	–	Rani et al., 2018
				10C	*E. coli*	BHI agar	37	24	6.17	–	–	
	*L. plantarum*		360.0	0.064 –1	P. aeruginosa	MHB broth	37	22–24	–	1	–	Mahdhi et al., 2017
	*L. plantarum*		360.0	0.064 –1	*S.* typhimurim	MHB broth	37	22–24	–	2	–	
	*L. rhamnosus* SHA111, 113,114,115,116,117	–	–	5	*E. coli*	LB agar	37	48	10.1–14.3	–	–	Riaz Rajoka et al., 2018
	*L. rhamnosus* SHA111, 113,114,115,116,117	–	–	5	*S.* Typhimurium	LB agar	37	48	7.8–13.1	–	–	
	*L. reuteri* SHA101	–	–	5	*S.* Typhimurium	LB agar	37	48	14	–	–	Riaz Rajoka et al., 2020
	*L. reuteri* SHA103	–	–	5	*E. coli*	LB agar	37	48	13.5	–	–	
	*L. vaginalis* SHA110	–	–	5	*S.* Typhimurium	LB agar	37	–	15	–	–	
	*L. vaginalis* SHA112	–	–	5	*E. coli*	LB agar	37	–	10.6	–	–	
	*L. brevis*	–	–	0.05-0.01 M	*E. coli*	N agar/N broth	28	48	13.72	0.008	–	
	*L. plantarum* C70	Ara, Man, Glu, Gal	380.0	5	*S.* Typhimurium	BHI	37	18	–	–	2.84	Ayyash et al., 2020a
			380.0	5	*E. coli* O157:H7	BHI	37	18	–	–	2.98	
*Lactococcus*	*Lc. garvieae* C47	Glu, Ara, Xyl	7300.0	5	*E. coli* O157:H7	N broth	37	24	–	–	2.77	Ayyash et al., 2020b
				5	*S.* Typhimurium	N broth	37	24	–	–	2.84	
	*Lc. lactis* F-mou	–	–	–	*E. coli*	MHB broth	37	–	–	17	–	Nehal et al., 2019
					*P. aeruginosa*	MHB broth	37	–	–	12.6	–	
					*P. mirabilis*	MHB broth	37	–	–	14.5	–	
					*A. baumannii*	MHB broth	37	–	–	12.5	–	
					*E. cloacae*	MHB broth	30	–	–	14.1	–	
*Streptococcus*	*S. thermophilus* GST-6	Glu and gal	–	10S	*E. coli*	BHI agar	37	24	12.1	4	–	Zhang et al., 2016
					*S.* Typhimurium	BHI agar	37	24	18.4	<2	–	
					*S. flexneri*	BHI agar	37	24	1.5	–	–	
	*S. thermophilus* GST-6	Glu and gal	–	10	*E. coli*	BHI agar	37	24	5.1	10	–	
					*S.* Typhimurium	BHI agar	37	24	7.6	6	–	
					*S. flexneri*	BHI agar	37	24	1.8	–	–	

The EPS produced by *L. kefiranofaciens* DN1 exhibited bactericidal and bacteriostatic activities against *S. enterica* serovar Enteritidis and *L. monocytogenes.* The effect was proportional to the concentration of the EPS (Jeong et al., 2017). Further, EPS from *Lactobacillus spp.*, showed a considerable antibacterial effect (inhibition zone > 10 mm) against *S. enterica* ATCC 43972 and *Micrococcus luteus* Ca6 (Trabelsi et al., 2017). EPS formed by *L. johnsonii* FI9785 was responsible for increasing the competitive inhibition of pathogens through surface hydrophobicity and auto-aggregation (Dertli et al., 2015). *Lactobacillus* EPSs have been reported to contain several functional groups, for instance, carbonyl, phosphate, and hydroxyl groups, which were suggested to play an essential role in exerting the antimicrobial and antioxidant effects of EPS (Riaz Rajoka et al., 2020). These polymers showed *in vitro* or *in vivo* inhibition of Gram-positive and Gram-negative pathogens with different degrees of resistance. The Gram-positive bacteria cell wall includes numerous structural components, which are essential components involved in the interaction between bacterial cells and various receptors on other surfaces (Al Kassaa et al., 2014). The cell surface of bacteria is a vital element in cell-to-cell and cell-to-host communications (Dertli et al., 2015). EPS have been shown to have a crucial role in modulating several features of the interaction between bifidobacteria and their host, such as reducing the immunological response toward commensal bacteria and providing protection against pathogens (Fanning et al., 2012).

Although the physicochemical properties of EPS are significantly associated with their bioactivities (Zhou et al., 2019), many of the studies reviewed in this report did not examine the electrical charge on the polymer. For instance, negatively charged EPS from the *Lactococcus lactis* F-mou strain revealed higher inhibitory action against Gram-positive than Gram-negative pathogens, with *B. cereus* ATCC 10702 demonstrating the greatest inhibition (Table 1; Nehal et al., 2019). The latter authors suggested that the negatively charged EPS (due to the sulfate group) could better interact with Gram-positive bacteria owing to their higher positive charge on the cell wall.

The findings of the studies shown in Table 1 are challenging to evaluate equitably. However, variations between studies may be explained by differences in the experimental approaches used, how the assessments of effectiveness, viability, dose format, inoculum size, and incubation duration were made, and differences related to growth conditions such as the type of medium used. These differences would be eliminated if only current approved standard methods published by the European Committee on Antimicrobial Susceptibility Testing (EUCAST^1^) and the Clinical and Laboratory Standards Institute (CLSI^2^) were employed.

**FIGURE 1:**
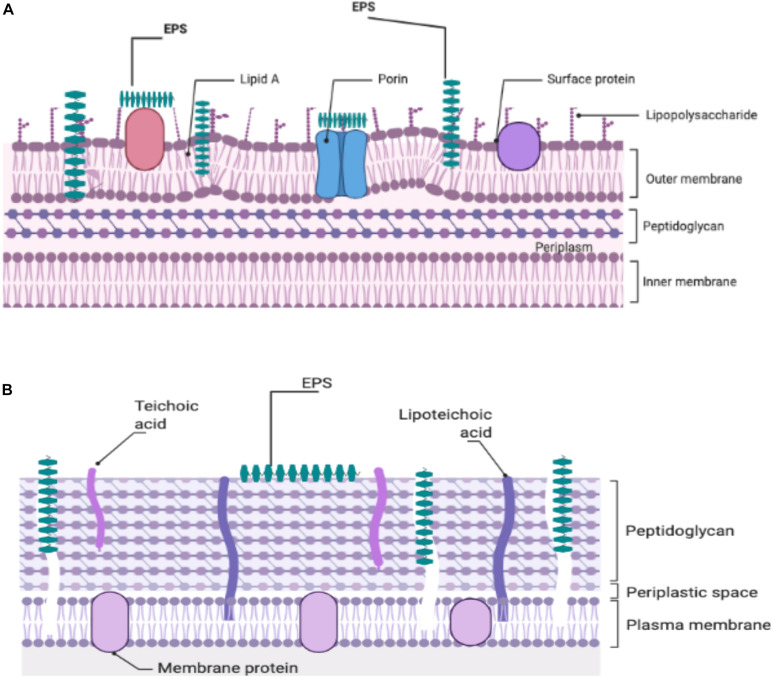
Illustration of the potential EPS-cell wall interactions of Gram-negative **(A)** and Gram-positive **(B)** responsible for the antibacterial effects of EPSs.

At the present time, there is no definitive mechanism to explain the antibacterial action of the EPS produced by the LAB against Gram-positive and -negative bacteria. Attempts to investigate a potential mechanism(s) to explain the observed antibacterial activity are continuing. It was thought that because EPS was able to disrupt the structure of the bacterial cell envelope, especially the peptidoglycan layer, it was proposed as a potential inhibitory mechanism (Sivasankar et al., 2018; Figure 1). The ability of bacterial EPSs like kefiran to interact with bacterial or eukaryotic cells led to the hypothesis that kefiran acts as a masking or decoy (Medrano et al., 2009). Thus, it may suggest that this action could block the receptors or channels on the outer membrane of the Gram-negative bacteria. Salachna et al. (2018) reported that EPS could facilitate accumulation of secondary metabolites in the growth media, which might adversely affect Gram-positive and -negative pathogens. It is likely that in some manner, functional groups in the structure of EPS interact with bacterial cell envelopes to yield antimicrobial activity (Zhou et al., 2019).

### Antifungal Activity and Potential Mechanism

*Lactobacillus spp.*, are the most commonly recognized LAB for their antagonistic potential (Graf et al., 2019). *Lactobacillus spp.*, have several possible ways of acting against *Candida* including a decrease of *Candida* adhesion through co-aggregation, immunomodulation of the host epithelial cells, and competition for binding sites (Allonsius et al., 2017).

The findings of recent investigations on the potential antifungal activities of EPSs produced by LAB are presented in Table 2. Examining the effect of wild-type *L. rhamnosus* GG, its mutant (lacking EPS), and the purified EPS against *Candida* revealed that the EPS layer might play an essential role in decreasing hyphal formation and during adhesion to vaginal epithelial cells (Allonsius et al., 2017). In the *in vitro* gut model, *L. rhamnosus* decreased hyphal elongation, a crucial virulence factor in *C. albicans-*induced cytotoxicity. Furthermore, in both *C. glabrata* and *C. albicans*, lectin-like adhesins recognizing glycans comprised of galactose residues have been described.

**TABLE 2:** Antifungal effects of the LAB-derived EPS.

Producing organism	Monosaccharides	Purified/Crude/Modified	Conc. mg/mL	Pathogens	*In vitro*/*In vivo*	Mode of action	References
*Lactococcus lactis F-mou*		Purified	16	*Candida albicans*	*in vitro*	Fungicidal effect	Nehal et al., 2019
*Lactobacillus rhamnosus GG*	Galactose-rich exopolysaccharides (EPS)	Purified	0.050, 0.1, 0.2	*Candida albicans* SC5314 *Candida glabrata* ATCC 2001	*in vitro*	Play an essential role in decreasing hyphal formation and during adhesion to vaginal epithelial cells	Allonsius et al., 2017

Negatively charged EPS produced by *Lc. lactis* F-mou exhibited antifungal activity against *C. albicans* (Table 2). The authors suggested that negatively charged EPS could provide better interaction with pathogens through its sulfate group (Nehal et al., 2019). The dextran formed by *Weissella confuse*, a probiotic strain isolated from Romanian yogurt, inhibited 70% of the biofilm formed by *C. albicans* SC5314. *L. rhamnosus* showed an ability to decrease *C. albicans* hyphal induction as well as biofilm formation through cell to cell interactions and the excretion of exo-metabolites (Matsubara et al., 2016). Also, *L. rhamnosus* GG protected the oral epithelial cells against harm caused by *C. albicans* through preventing fungal adhesion and reducing available nutrients (Mailänder-Sánchez et al., 2017).

Exopolysaccharide produced by *Lactobacillus* strains revealed a remarkable antifungal effects against *C. pelliculosa* compared with Gram-positive (*L. innocua, S. aureus*, and *Micrococcus luteus*) and Gram negative bacteria (*P. aeruginosa* and *E. coli*). Furthermore, variations in inhibitory activity among strains indicated a strain-dependent rather than a species-dependent effect (Abouloifa et al., 2019).

### Anti-biofilm Activity and Potential Mechanism

The term biofilm refers to an arrangement of bacterial cells to form a community embedded in a self-formed extracellular polymeric matrix adherent to an inert or living surface (Vu et al., 2009; Simões et al., 2010). Pathogenic bacteria have been reported to form biofilms and get attached as a response to environmental stresses and to protect themselves against host antagonistic activity (Kim et al., 2009). Biofilms produced by pathogenic bacteria are recognized as a substantial source of chronic and acute infections, mainly owing to their capability of persisting on surfaces and in medical devices (Kim et al., 2009). Also, they are considered a significant threat to food safety because of their resistance to conventional decontamination treatments. Research examining biofilm control, eradication, or prevention has gained increasing attention recently because of biofilm effects on human health. The biofilm matrix produced by the Gram-negative pathogen *Pseudomonas aeruginosa* is composed of a viscous mixture of EPS (containing the non-mucoid polysaccharides Psl and Pel, plus alginate), proteins such as CdrA and extracellular DNA (eDNA) (Maunders and Welch, 2017).

While in the oral cavity and respiratory tract, these adherent structured microbial communities (biofilms) are responsible for dental caries, periodontitis, and respiratory infections; in the gastrointestinal tract (GI), these biofilm communities containing beneficial organisms such as lactobacilli may play an essential role as protective agents (Aoudia et al., 2016). Biofilm formation is a complex and dynamic process comprising five steps including: (a) initial attachment, (b) irreversible attachment, (c) initial growth of the biofilm structure, (d) maturation, and (e) dispersion (Spanò et al., 2016). Once the cells start to produce the extracellular polymeric substances, the adhesion to surfaces becomes irreversible.

Recent findings on the potential of LAB-derived EPS to reduce or prevent biofilm formation by pathogenic bacteria and thereby control or prevent infectious diseases caused by these pathogens are presented in Table 3. EPS produced by *L. plantarum* YW32 showed the ability to suppress biofilm formation by Gram-positive and -negative pathogens. Wang et al. (2015) suggested that EPS might interfere with biofilm activity by modifying bacterial cell surfaces, hindering the initial attachment of bacterial cells to the surface, or by down-regulating gene expression involved in biofilm formation by acting as a signaling molecule (Wang et al., 2015).

**TABLE 3:** Antibiofilm activity of LAB-derived EPS against bacterial pathogens.

Genus	Specie/Strain	Monosaccharides	Mw (kDa)	Conc. mg/mL	Target Pathogens	Growth medium	Test Temp (°C)	Inc. Time (h)	Inhibition (%)	References
**Against Gram-positive**										
*Enterococcus*	*E. faecium* K1	Man, gluc, gal	–	4	*E. faecalis*	TSB/BHI	37	18	75.85	Bhat and Bajaj, 2018
				4	*B. cereus*	TSB/BHI	37	18	65.56	
				4	*S. aureus*	TSB/BHI	37	18	61.45	
				4	*B. subtilis*,	TSB/BHI	37	18	55.5	
*Lactobacillus*	*L. plantarum* WLPL04	Xyl, Glu, Gal	66.1	5	*S. aureus* CMCC26003	LB broth	37	24	∼ 43	Liu et al., 2017
	*L. gasseri* FR4	Glu, man, gal, rha, fuc	186.0	4	*L. monocytogenes* MTCC657	LB broth	37	24	56	Rani et al., 2018
				4	*S. aureus* MTCC 3160	LB broth	37	24	∼ 27	
				4	*E. faecalis* MTCC 439	LB broth	37	24	19.2	
	*L. plantarum* YW32	Man, fru, gal, glu	103.0	5	*S. aureus* AC1	LB broth	37	24	45.13	Wang et al., 2015
	*L. casei* NA-2	Rha, glu, man	–	5	*B. cereus* CICC 21261	NB broth	37	24	95.5	Xu et al., 2020a
				5	*S. aureus* CGMCC 1.291	TSB broth	37	12	30.2	
	*L. paracasei* M7	Man, glu, gal	–	4	*E. faecalis*	TSB/BHI	37	18	64.27	Bhat and Bajaj, 2018
				4	*B. subtilis*	TSB/BHI	37	18	63.84	
				4	*B. cereus*	TSB/BHI	37	18	62.89	
				4	*S. aureus*	TSB/BHI	37	18	61.45	
	*L. plantarum*	–	36.0	0.256	*S. aureus* ATCC 25923	TSB-YE brot	37	24	–	Mahdhi et al., 2017
				0.256	*L. monocytogenes* ATCC 19115	TSB-YE brot	37	24	–	
	*L. rhamnosus* SHA117	–	–	5	*S. petrasii* subsp. *pragensis*	LB broth	37	24	70.5	Riaz Rajoka et al., 2018
				5	*S. petrasii* subsp. *pragensis*	LB broth	37	24	61.1	
	*L. coryniformis* NA-3	Rha, man, gal, glu	8600.0	0.5	*B. cereus*	Nutrient Broth	37	24	80	Xu et al., 2020b
**Against Gram-Negative**										
*Enterococcus*	*E. faecium* K1	Man, gluc, gal	–	4	*Klebsiella sp.*	TSB/BHI	37	18	55.25	Bhat and Bajaj, 2018
				4	*P. aeruginosa*	TSB/BHI	37	18	55.12	
*Lactobacillus*	*L. plantarum* WLPL04	Xyl, Glu, Gal	66.1	5	*P. aeruginosa* CMCC10104	LB broth	37	24	47.02	Liu et al., 2017
				5	*E. coli* O157:H7	LB broth	37	24	25.8	
	*L. gasseri* FR4	Glu, man, gal, rha, fuc	186.0	4	*E. coli* MTCC 2622	LB broth	37	24	∼27	Rani et al., 2018
	*L. plantarum* YW32	Man, fru, gal, glu	103.0	5	*S.* flexneri CMCC (B)	LB broth	37	24	44.67	Wang et al., 2015
				5	*S.* Typhimurium S50333	LB broth	37	24	44.04	
				5	*E. coli* O157	LB broth	37	24	12.7	
	*L. casei* NA-2	Rha, glu man	–	5	*S.* Typhimurium ATCC 14028	TSB broth	37	24	12.1	Xu et al., 2020a
				5	*E. coli* O157:H7 CICC 10907	TSB broth	37	24	16.9	
	*L. plantarum* WLPL04	Xyl, Glu, Gal	66.1	5	*S.* Typhimurium ATCC1331	LB broth	37	24	∼28	Liu et al., 2017
	*L. paracasei* M9	Man, glu gal	–	4	*Klebsiella* sp.	TSB/BHI	37	18	59.42	Bhat and Bajaj, 2019
	*L. paracasei* M10	Man, glu gal	–	4	*P. aeruginosa*	TSB/BHI	37	18	58.88	
	*L. plantarum*	–	36.0	0.256	*P. aeruginosa* ATCC 33787	TSB-YE brot	37	24	–	Mahdhi et al., 2017
				0.256	*S.* Typhimurium ATCC 14028	TSB-YE brot	37	24	–	
	*L. rhamnosus* SHA117	–	–	5	*E. coli*	LB broth	37	24	28.4	Riaz Rajoka et al., 2018
				5	*S.* Typhimurium	LB broth	37	24	63	
				5	*E. coli*	LB broth	37	24	17.6	
				5	*S.* Typhimurium	LB broth	37	24	51.1	
	*L. coryniformis* NA-3	Rha, man, gal, glu	8600.0	0.5	*S.* Typhimurium	TSB broth	37	24	40	Xu et al., 2020b

The EPS produced by *L. casei* NA-2 inhibited biofilm formation by *B. cereus* by more than 95.5%, followed by *S. aureus* (30.2%), *S.* Typhimurium (12.1%), *E. coli* (16.9%), as shown in Table 3. Rani et al. (2018) reported that the EPS of *L. gasseri* FR4 showed the highest antibiofilm effect against *L. monocytogenes* MTCC 657 (56% inhibition) and the lowest effect (19.2%) against *Enterococcus faecalis.* Additionally, the EPS of *L. plantarum* WLPL04 inhibited *P. aeruginosa* biofilm formation by 47.02% and that of *E. coli* O157:H7 by 25.82% (Liu et al., 2017). Bhat and Bajaj (2018) found that EPS from *E. faecium* K1 inhibited biofilm formation by a wide range of bacterial pathogens, including *E. faecalis, B. cereus, S. aureus, Bacillus subtilis, Klebsiella* spp., and *P. aeruginosa* (Table 3). The results presented in Table 3 indicate that EPS produced by LAB generally and *Lactobacillus* strains, in particular, have a wide range of anti-biofilm effects against biofilm-forming pathogens. Thus, EPS could be a possible candidate for use as a food-grade additive in the food industry for controlling or preventing the growth of biofilm-forming bacteria. It is possible to spray a solution containing EPS produced by Lactobacillus strains. EPSs formed by some *Lactobacillus* strains showed the ability to decrease colonization by microbial pathogens, thus supporting host health through enhancing the immune response (Fanning et al., 2012). EPS showed the ability to inhibit *E. coli* O157:H7 adhesion to human colorectal adenocarcinoma (HT-29) cells in competition, replacement, and inhibition tests. Moreover, the EPS displayed potent inhibition of bacterial pathogens in biofilms formed by *P. aeruginosa* CMCC10104, *E. coli* O157:H7, *S.* Typhimurium ATCC13311, and *S. aureus* CMCC26003 (Liu et al., 2017).

The disruptive effect of EPS produced by *L. coryniformis* NA-3 on pre-formed *S.* Typhimurium and *B. cereus* biofilms was greatest among all those tested and was reported to be 80 and 90%, respectively (Xu et al., 2020b). EPS formed by *L. fermentum* LB-69 isolated from children’s feces inhibited biofilm formation by *B. cereus* RSKK 863. It has been proposed that the weakening of cell wall integrity due to cell surface alterations or decreasing cell-to-cell interactions could have been responsible for inhibition of the initial auto-aggregation and attachment of bacterial cells (Kanmani et al., 2013).

The enormous variations apparent among investigations may be caused by the utilization of different experimental conditions (Table 3). Contradictory results could also arise following the use of different non-standardized methodologies such as inoculum preparation procedures, growth medium, inoculum size, incubation conditions and duration, varying dose, assessment of efficacy, and especially due to the variation in selection of strains. It is well known that the impact of a probiotic is strain-specific; different strains can have a different impact on a pathogenic biofilm because molecular signaling in bacteria tends to be remarkably strain-dependent. Thus, standard procedures must be developed and followed to assure implementation of an essentially identical experimental approach in order to legitimately permit comparison of results from different studies.

One of the suggested approaches to prevent or eliminate biofilm formation is to block the quorum sensing (QS)-mediated systems which will hamper the early adhesion and subsequent formation of mature structures (Paluch et al., 2020). The QS system provides a means by which cell-to-cell bacterial communication occurs and is essential for biofilm formation and maintenance of its structural integrity. In addition, QS regulates many essential social behaviors such as motility, bioluminescence, virulence, sporulation, antibiotic production, and genetic (transformation) competence (Reuter et al., 2016; Barzegari et al., 2020). The system relies on the production, release, and detection of extracellular chemical signaling molecules known as auto-inducers (Whiteley et al., 2017; Barzegari et al., 2020). *S. aureus* is a representative Gram-positive bacterium possessing the QS mechanism, but it also has an additional *agr* system that controls the formation of virulence factors such as exotoxins or biofilms (LaSarre and Federle, 2013; Paluch et al., 2020).

Gram-negative bacteria use *N*-acylated homoserine lactones (AHLs), synthesized by a LuxI type enzyme, as self-inducers. The bacteria typically produce the signals (AHLs) on a continuous basis, beginning at a low concentration and building up in the local environment as the population density rises (Abisado et al., 2018). When they reach a suitable threshold concentration, the LuxR receptor protein is activated, and transcription of related genes follows (Parashar et al., 2013; Alayande et al., 2018). As a representative model of the QS system in Gram-negative bacteria, *P. aeruginosa* has two pairs of LuxI/LuxR homologs: LasI/LasR as well as RhlI/RhlR. The QS system of this bacterium coordinates the formation of biofilms and the expression of several virulence factors, including alkaline phosphatase, exotoxin A, protease, and elastase (Paluch et al., 2020).

Regarding the *in vitro* situation, EPSs produced by LAB are identified as foreign molecules by bacterial pathogens. As a result of their large size or charge, they are not able to pass or be transported into pathogens and consequently cannot exert their effect inside the cell. Thus, one possible way the antimicrobial effect of EPS occurs is through their ability to interfere with the biofilm signaling molecules or by blocking the glycocalyx receptors at the surface of the pathogen, causing quorum-quenching; and as a result, impeding the formation of biofilms (Spanò et al., 2016; Zhou et al., 2019). In contrast, the antimicrobial mechanism is considerably more sophisticated, associating with not only the developing biofilm but also modifying competitive relationships and minimizing immunological responses.

Exopolysaccharidesproduction by some biofilm-forming, probiotic LAB could be another possible mechanism for preventing biofilm formation by pathogenic bacteria (Salas-Jara et al., 2016). Probiotic LAB biofilms can enhance their own colonization and stability in the host mucosa and thus inhibit bacterial pathogen colonization. However, only a few *Lactobacillus* strains, for instance, *L. fermentum, L. plantarum*, *L. rhamnosus*, and *L. reuteri* have been reported to form biofilms on abiotic surfaces such as polystyrene or glass (Salas-Jara et al., 2016). Biofilm formation by probiotic LAB is recognized as a promising strategy to retard biofilm formation by pathogenic bacteria since they can successfully compete with bacterial pathogens for space and nutrients (Barzegari et al., 2020).

Supernatants of numerous marine bacterial strains displayed anti-biofilm activities with their active compounds ranging from furanones to complex polysaccharides described as significant potential QS inhibitors (Spanò et al., 2016). EPS may directly adhere to intestinal mucus and thus competitively inhibit both probiotic and pathogen adhesion or binding to the intestinal epithelium. Further, the resulting enhanced pathogen interaction with EPS may facilitate pathogen retention in the intestinal lumen and subsequent clearance (Lynch et al., 2018). Kefiran, a branched polysaccharide, has been suggested to play a masking role in preventing bacterial cells and their toxins from binding to the EPS instead of the receptors on the enterocyte surface (Medrano et al., 2009). However, the same protective activity was not detected with dextran, signifying the importance of composition and structure of the EPS in facilitating these effects (Lynch et al., 2018).

### Antiviral Activity (*in vitro* and *in vivo*)

It is of interest that because probiotic LAB and their metabolic products have been shown to have an antiviral activity that they might represent a promising approach for treating viral diseases (Ryan et al., 2015; Olaimat et al., 2020). Probiotic bacteria and their metabolites, including EPS, may interfere with virus infection by modifying the state of cells *via* stimulating both an innate and adaptive immune response (Sundararaman et al., 2020). Numerous studies concerning the biological significance of probiotics on host immunity proposed that they have an essential role in regulating the functions of mucosal immune cells and intestinal epithelial cells (IECs). Production of antiviral inhibitory substances, stimulation of the immune system, and direct interaction with viruses are some of the proposed mechanisms utilized by LAB for exerting their antiviral activities (Al Kassaa et al., 2014).

The data from recent studies on the potential antiviral activities of LAB-derived EPS are shown in Table 4. *In vitro*, EPS from *L. plantarum* LRCC5310 showed a strong anti-rotavirus effect, particularly against extracellular rotaviruses. Additionally, *in vitro*, it showed a high adhesion rate, consequently interfering with the attachment of the rotavirus to MA104 cells. *In vivo*, EPS produced by *L. plantarum* LRCC5310 decreased the period of diarrhea, reduced rotavirus replication in the intestine, and decreased the recovery time of young mice (Kim et al., 2018). EPS could improve vaccine-induced protection as new adjuvant systems. Also, they could provide a robust designated immune response, particularly against challenging viral pathogens including novel influenza pandemic strains (H1N1) and highly mutable viruses such as hepatitis C and AIDS (Moscovici, 2015). The EPS formed by strains belonging to the genera *Pediococcus, Leuconostoc*, and *Lactobacillus* showed low virucidal activity and reduced the HAdV-5 infectivity to 85%. Additionally, among the investigated EPS, EPS 26a formed by *Lactobacillus spp.*, showed anti-adenovirus activity. Furthermore, the treatment of cells with the EPS showed a complete (100%) adsorption of the virus and suppressed the formation as well as the release of infectious HAdV-5 particles, demonstrating that 26a had exceptional anti-HAdV-5 activity and had the potential for being used as an anti-adenoviral agent (Biliavska et al., 2019). Mao et al. (2016) reported that *Lactobacillus spp.*, could reduce diarrhea and hinder the replication of rotavirus *via* enhancing intestinal barrier function. *L. plantarum* LRCC5310 has been suggested to protect the intestinal mucosal barrier through reducing shedding and other damage caused by virus infection (Kim et al., 2018). The most recognized probiotic strain, *L. rhamnosus* GG, was found to have a positive activity against gastroenteritis caused by rotavirus in hospitalized infants and children (Sindhu et al., 2014).

**TABLE 4:** Antiviral activities of LAB-derived EPS.

Producing bacterium	Purified/Crude/Modified	Conc. mg/mL	Pathogens	*In vitro*/*In vivo*	Mode of action	References
*Pediococcus* spp., *Leuconostoc* spp., *Lactobacillus spp.*,	Purified	0.02, 0.1, 0.5	Adenovirus type 5 (HAdV-5)	*in vitro*	Only EPS 26a from *Lactobacillus* sp. obstructed HAdV-5 reproduction	Biliavska et al., 2019
*Lactobacillus plantarum* LRCC5310	Purified	1.95	Rotavirus	*in vitro*	Interfering with the rotaviral attachment to cells *in vitro*	Kim et al., 2018
*Lactobacillus plantarum* N4(Lp)	Purified	1/2, 1/4, 1/8, 1/16	Transmissible Gastroenteritis Virus (TGEV)	*in vitro*	Inhibitory effect on TGEV	Yan et al., 2020
*Lactobacillus delbrueckii* OLL1073R-1	Purified neutral and acidic EPS	0.1	DsRNA similar to that found in many viruses	*in vitro*	EPSs treatment induced the expression of IFN-α and IFN-β in PIE cells as well as the antiviral factors MxA and RNase L	Kanmani et al., 2018b
*Lactobacillus delbrueckii* subsp. *delbrueckii* TUA4408L	Purified neutral and acidic EPS	0.1	Rotavirus	*in vitro*	Enhanced expression of the antiviral factors interferon (IFN)-β, Myxovirus resistance gene A (MxA) and RNaseL	Kanmani et al., 2018a
*Lactobacillus plantarum* LRCC5310	Purified	1	Rotavirus	*in vivo*	Decreased the duration of diarrhea, restricted PIE lesions, reduced rotavirus replication in the intestine, and reduced the time to recovery	Kim et al., 2018

## Antimicrobial Activity of Modified Lab-Derived EPS

Modifications of EPS by group substitution have been reported to impact their physicochemical and biological activities (Zhou et al., 2019). The substitutional modifications include sulfonation, phosphorylation, acetylation, and selenylation. Sulfation has been recognized to be advantageous to numerous biological activities, including anti-coagulation, antitumor action, antimicrobial and immunity-regulation activities (Zhou et al., 2019). Among investigated modified EPSs, sulfated EPSs have revealed potent antiviral activity. They were shown to be effective in inhibiting the virus-cell attachment; also, they exhibited antiviral activity against several types of viruses such as human cytomegalovirus, influenza virus, herpes simplex virus, and hepatitis B virus (Oh et al., 2010). Sulfated EPS from *L. plantarum* ZDY2013 and *Streptococcus thermophilus* ASCC1275 exhibited a greater antimicrobial effect against various Gram-positive and harmful pathogens than the non-sulfated ones (Li and Shah, 2014; Zhang et al., 2016). The inhibitory effect could have been due to the disruption of the biofilm-mediating signaling pathway or from injury to the cell membrane (Li and Shah, 2014; Zhang et al., 2016). Also, sulfated EPS showed a better ability to protect cells against enterotoxin-induced cytotoxicity (Zhang et al., 2016). sulfated EPS from *Streptococcus thermophilus* GST-6 displayed more robust inhibitory activity than did the non- sulfated EPS against bacterial pathogens, including *E. coli*, *S.* Typhimurium, and *S. aureus* (Zhang et al., 2016). It has been proposed that negatively charged EPS is capable of acting as a mild stimulator to various immune cells, while neutral EPS can suppress the immune response. Thus, the neutral EPS could protect the EPS-producing bacteria against the immune system of the host (Hidalgo-Cantabrana et al., 2012).

Physicochemical properties of EPS are associated with their immune-modulating ability. It has been reported that substitution with a highly negatively charged group enhances the immune-regulatory effect of EPS polymers (Hidalgo-Cantabrana et al., 2012). It is likely that sulfate, phosphate and/or carboxylate repeat groups are essential forthe immune-regulatory activity of EPS. For instance, acidic hetero-EPS (HeEPS) characterized by their negatively-charged phosphate group were better stimulators of the immune response than uncharged EPS (Hidalgo-Cantabrana et al., 2012). Chemical phosphorylation of the dextran (α-glucan HoEPS) produced by *Leuconotoc mesenteroides* enhanced the production of lymphocyte subsets from the murine spleen and increased the gene expression of IL-10 and IFNc (Hidalgo-Cantabrana et al., 2012). Thus, the importance of the phosphate group in immune stimulation was demonstrated.

## Immunomodulation by Lab-Derived EPS as Antimicrobial Action

### Antibacterial Immune Response Activity

Recent studies have revealed that LAB with immunomodulatory competencies exert their positive effects *via* several substances, for instance, the cell wall, its peptidoglycan, and through EPS that can interact with specific receptors on the host cells (Laiño et al., 2016). The IECs or dendritic cells (DCs) interact with the host gastrointestinal microbiota *via* their pattern recognition receptors (PRRs) that can sense microbe-associated molecular patterns (MAMPs) (Caggianiello et al., 2016). The communication between PRRs and MAMPs causes stimulation of signaling pathways, which regulate the type of the molecular response (for instance, antimicrobial action or stimulation, often involving chemokines or cytokines) targeting a particular microorganism (Lebeer et al., 2010). Glycans have a significant role in this process, probably by modifying ligand-receptor communications and aiding in differentiating among the MAMPs of LAB, commensals, and pathogens (Lebeer et al., 2010; Round et al., 2011).

Regarding their ability to induce immunoglobulin A (IgA), *Leuconostoc* (*Leu.*) *mesenteroides* NTM048 EPS was suggested to be effective against pathogenic microbes (Matsuzaki et al., 2014, 2015). It was found that IgA, the most copious immunoglobulin (Ig) isotype in epithelial mucus, can capture antigens, consequently stopping their binding to cell surface receptors (Matsuzaki et al., 2014). This type of action was seen in the decrease in motility of *Salmonella* spp. and IgA mediation of cholera toxin neutralization (Forbes et al., 2008). The EPS from *Leu. mesenteroides* NTM048 has been shown to enhance the mucosal barrier defense *via* simulation of IgA secretion, which sequentially induces the mucosal immune system of the host. *In vitro*, the NTM048 EPS stimulated both total and antigen-specific IgA production as well as Type 1 T helper (Th1) and Type 2 (Th2) cell-mediated responses in splenocytes (Matsuzaki et al., 2015). EPS produced by *L. rhamnosus* KL37 showed an ability to stimulate macrophage production of cytokines, TNF-α, IL-6, and IL-12 (Ciszek-Lenda et al., 2011).

*Bifidobacterium breve* UCC2003 strains producing surface-linked EPS failed to stimulate a potent immune response compared with their EPS-lacking mutants. EPS production was seen to be associated with the avoidance of adaptive B-cell responses. Additionally, the presence of EPS-producing *B. breve* decreased the levels of *Citrobacter rodentium* colonization of the murine gut (Fanning et al., 2012).

The molecular weight of EPSs has been shown to have a significant impact on their bioactivities, but not all studies report the molecular weight of the EPS examined (Table 1). Production of high molecular weight EPS by a mutant of *B. animalis* subsp. *lactis* decreased its ability to adhere to human IECs and weakened biofilm formation on abiotic surfaces (Castro-Bravo et al., 2017). Thus, it may be concluded that high molecular weight EPS may negatively impact intestinal colonization. Heter-EPS (HeEPS) with high molecular weight may act as suppressors of the immune response, thus helping the producing bacteria to evade the immune response of the host (Hidalgo-Cantabrana et al., 2012). EPSs with smaller sizes are suggested to have the ability to act as mild stimulators of various immune cells, while polymers with bigger sizes (close to 10^6^ Da) could have a suppressive effect or even weaken an extreme response (Hidalgo-Cantabrana et al., 2012). The presence of impurities or contamination of EPS with other MAMPs, for instance, lipopolysaccharides lipoproteins, lipoteichoic acid, or other cell wall components that could stimulate the immune response in IECs or DCs *via* their PRRs, could be a significant factor contributing to misleading conclusions from *in vitro* antimicrobial immunological studies of EPSs.

### Antiviral Immune Response Activities

It is proposed that the mechanism by which LAB exert their antiviral effect involves direct probiotic–virus interaction, production of inhibitory antiviral metabolites, and stimulation of the immune system (Al Kassaa et al., 2014). EPS as well as several other cellular components including DNA, RNA, and bacterial cell wall components (e.g., lipoteichoic acid, teichoic acids, peptidoglycan, pellicle, and surface layer proteins) could modulate the innate antiviral immune response (Quinteiro-Filho et al., 2015; Laiño et al., 2016). EPSs formed by probiotic LAB could be potential candidates in antiviral therapy for preventing or treating viral infections in both humans and animals with significant effectiveness (Oh et al., 2010). In vaccine preparations, EPSs could be utilized as antigen carriers or as antigens themselves against some viral pathogens such as influenza H1N1 and human hepatitis B. Also, it has been reported the utilization of EPS could enhance the immunogenicity of the hepatitis B vaccine (Sharma et al., 2009; Mocanu et al., 2011; Li et al., 2014; Moscovici, 2015). Various extracellular EPSs produced by particular probiotic LAB have been proposed to have immunomodulatory effects that could enhance the expression of the antiviral factors RNase L and MX1 in porcine IECs (Kanmani et al., 2018b). EPS produced by *L. delbrueckii* OLL1073R-1 showed the ability to enhance antiviral immunity, particularly in the systemic and respiratory compartments (Kanmani et al., 2018b).

*In vivo*, oral administration of yogurt fermented with EPS-producing *L. bulgaricus* OLL1073R-1 resulted in a considerable reduction of influenza virus titer and a large increase of virus immunoglobulin IgG_1_ and IgAa. Additionally, natural killer (NK) cell activity of splenocytes increased considerably in two groups of mice treated with EPS. Furthermore, oral administration of neutral or acidic EPS prior to intranasal infection prolonged survival in the acidic EPS-treated group, but not in the neutral EPS-treated group (Nagai et al., 2011). The EPS from *L. delbrueckii* TUA4408L stimulates the signaling pathway of interferon regulatory factor-3 and nuclear factor-_*k*_B which boosts the immune response through increasing the expression of the antiviral factor interferon-β, myxovirus resistance gene A, and RNase L (Kanmani et al., 2018a). The EPS from *L. delbrueckii* OLL1073R1 exhibited the ability to modulate the innate immune system in porcine IECs *via* decreasing the expression of IL-6 and the pro-inflammatory chemokine (Kanmani et al., 2018b).

*In vitro*, EPS from *L. delbrueckii* OLL1073R-1 showed a substantial ability to induce an increase in the expression of IFN-α, IFN-β, ISGs MxA, and RNase L in IECs when stimulated with poly (I:C). MxA proteins have been reported to have an inhibitory activity that could protect against infection by several viruses (Xiao et al., 2013). Additionally, RNase L damages viral RNA and initiates cytoplasmic RIG-I/MDA5 receptors, which induce an increase in type I IFN production and increase the apoptosis of infected cells (Chakrabarti et al., 2011). *In vivo*, administration of curdlan sulfate to mice immunized with recombinant hepatitis B surface protein (HBsAg) considerably boosted the macrophages and DCs in the spleen and improved the numbers of antigen-specific CD8 + and CD4 + cells. In addition, curdlan sulfate enhanced the expression of IFN-γ, decreased the expression of IL-4, and increased HBsAg-specific antibodies IgG2a/IgG1 within the anti-HBsAg antibodies in mice immunized with HBsAg plus curdlan sulfate, compared with those receiving only HBsAg (Li et al., 2014). Additional investigations of the underlying mechanisms are needed in order to enable the successful development of LAB-derived EPS as a potential antiviral therapeutic agent.

**FIGURE 2:**
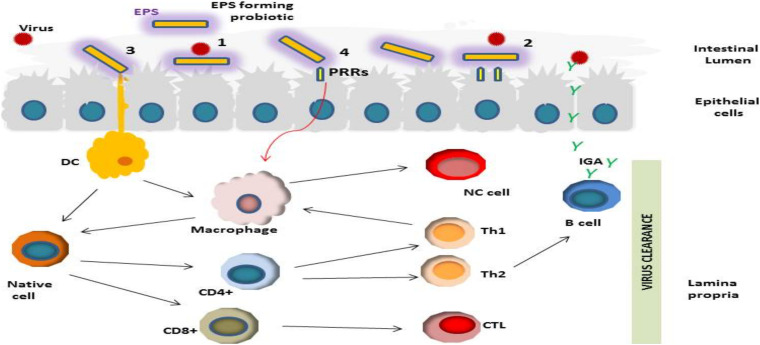
Schematic representation of different potential antiviral activities of LAB-derived EPSs including; (1) Prevention of the viral infection through direct binding of viral particles; (2) Obstruction *via* shielding or masking virus pattern recognition receptor (PRRs) sites; (3) and (4) Stimulation of the immune response in mucosal epithelial and dendritic cells (DC) through their PRRs, resulting in induction of various cytokine production which consequently could modulate antiviral effects by activation of CD+ T lymphocyte differentiation into cytotoxic T lymphocytes; induction of macrophages, stimulation of antiviral activity *via* phagocytes by activation of Th1, induction of B cell proliferation and viral neutralization activity *via* secretion of antibodies.

Based on recent outcomes reported in the literature, we suggest a hypothetical mechanism (s) of the antiviral actions of LAB-derived EPS (Figure 2). The first step in the viral infection process is preventing virus adsorption to the host cell (Oh et al., 2010; Yan et al., 2020). EPS may prevent viral infection *via* blockage of virus adsorption onto the host cells through interacting either with virus particles or with the host cell (Liu et al., 2004). Probiotic bacterial metabolites such as EPS may indirectly hinder the virus by modifying the state of cells, stimulating innate and adaptive immunity (Sundararaman et al., 2020).

*In vitro*, cytokine responses to pure EPS and EPS producing and non-EPS producing LAB strains have been compared by numerous studies (Table 4). The purity level of the EPS was unclear. Additionally, in most studies, standard procedures were followed for EPS isolation, which may favor the co-precipitation of other bacterial components that possess immunogenic activities (Castro-Bravo et al., 2018). Finally, most investigators did not test the final product (purified EPS) for contamination with other microbe-associated molecular patterns (MAMPs), for instance, lipoproteins or lipoteichoic acid, which could stimulate an immune response in cells *via* their PRRs.

## Assessing Methods of the Antimicrobial Activities of EPS

Recently, LAB and probiotic-derived EPS have been getting much consideration as potential novel antimicrobial agents. Hence, several methods are commonly utilized to screen or evaluate the potential *in vitro* antimicrobial effect of crude extracted or purified EPS. The most recognized ones are the disk diffusion and dilution method (broth or agar). For these methods, there are several established and approved standards that are periodically updated and published by the European Committee on Antimicrobial Susceptibility Testing (EUCAST) and the Clinical and Laboratory Standards Institute (CLSI). The CLSI standard M02 is used for testing bacteria by the disk diffusion method, while CLSI standard M07 is applied for broth microdilution, broth macrodilution, and agar dilution. These testing methods give reliable results when implemented in accordance with the procedures described by the CLSI or by the commercial manufacturers of the test materials.

Disk diffusion is a low cost and straight-forward method which can be used for screening of many antimicrobial substrates and microorganisms. However, a drawback is that this method is not suitable for determining the minimum inhibitory concentration (MIC) since it is difficult to measure the quantity of the antimicrobial substrate that diffuses into the agar medium (Balouiri et al., 2016). For MIC value determinations, dilution methods are the most applicable, either agar or broth (macro or microdilution), as they can quantitatively estimate the *in vitro* antimicrobial effect against bacteria or fungi.

When reviewing the results of the published studies on the *in vitro* antimicrobial activities of EPSs (Table 1), the comparison among studies is often difficult, owing to the utilization of different non-standardized methodologies, including inoculum preparation procedures, growth medium, inoculum size, incubation conditions, and duration. It is well recognized that some factors such as the type of growth medium, inoculum preparation method, inoculum size, and the incubation duration can impact the MIC values (Balouiri et al., 2016). Thus, it is essential that the recommendations of EUCAST and CLSI be followed to guarantee a consistent experimental approach and allow the comparison of results from different studies.

For an in-depth investigation of the antimicrobial activity of EPS, other methods such as flow cytofluorometric and time-kill tests are suggested which have the advantages of getting information about the nature of the inhibitory activity, whether bacteriostatic or bactericidal, time-dependent or concentration-dependent and the nature of the damage caused to the examined microorganism. Future studies should avoid discrepancies in analytical techniques by making comparisons using consistent experimental approaches.

## Conclusion

Exopolysaccharidesproduced by LAB play an essential role in affecting the communications of these bacteria with the diverse microbial community. They are recognized as one of the suggested tools by which LAB facilitate some of their health-promoting effects. Recent studies revealed the importance of their antagonistic role to microbial pathogens, including viruses, bacteria, and fungi. EPSs exhibited potential for the treatment of several conditions, including viral and bacterial infections, enhancing vaccination responses, and minimizing allergy. The EPSs production by some biofilm-forming probiotic LAB could be a promising potential mechanism for preventing biofilm formation by pathogenic bacteria and inhibiting their colonization of device surfaces and susceptible individuals.

It has been reported that the EPS chain-length and molecular weight (Mw) influence its biological activities such the antimicrobial activities (Zhou et al., 2019). The larger EPS molecules creates high viscosity by changing its structure from double helical forms to macroscopic gels. Consequently, the antimicrobial activity will be improved as a result of the viscosity-related biological activity (Zhou et al., 2019). On the other hand, EPSs with low Mw and extra negative charge may improve the immunological effects in host (Xu et al., 2019). The presence of uronic and sulfate groups could improve the biological activities (e.g., anticancer and antimicrobial) of the EPS (Xu et al., 2019). The mechanisms by which EPSs act on microbial pathogens and the host immune system are very complex and still not fully understood. Thus, further investigations are necessary, particularly into the mechanism by which EPSs exert their effects as well as the exact factors (for instance, molecular, structural, and active groups) that act on microbial cells or stimulate epithelial cells. Prospective studies of structural-immune interactions should avoid the current variations seen in analytical techniques and immunological models by performing comparisons using consistent experimental approaches.

A better understanding of the underlying mechanism(s) could result in the development of EPS or EPS-based antivirus, antibacterial, or antifungal therapeutic agents. However, the impurity of prepared EPS contaminated with other microbe-associated molecular patterns (MAMPs), for instance, lipoproteins, lipoteichoic acid, or other cell wall components that could stimulate an immune response in epithelial cells (IECs) or DCs *via* their PRRs, could be a significant factor causing misleading results from *in vitro* antimicrobial immunological studies of EPSs. Thus, testing EPS for impurity is critical for such types of investigation.

Analysis of published literature indicates that applications for the antimicrobial activity of EPS are encouraging. It is well-established that some factors such as the type of growth medium, inoculum preparation method, inoculum size, and the incubation duration can affect MIC values. Moreover, the EPS preparation and purification, nature (HoEPS or HeEPS), molecular weight, monosaccharides composition, and functional groups are also crucial factors that affect the biological activities, including the antimicrobial activity. The procedures and recommendations of EUCAST and CLSI are highly recommended to be followed to assure consistent experimental approaches enabling comparison of results from different studies. More in-depth investigations of the antimicrobial activity of EPS are crucial. Therefore other methods such as flow cytofluorometric and the time-kill tests could offer important information about the nature of the inhibitory activity, whether bacteriostatic or bactericidal, time-dependent or concentration-dependent, and the nature of the damage caused to the cells examined.

## Author Contributions

MA conceptualized and designed the manuscript. AA and MA wrote the first draft. AA, AO, and TO wrote sections of the manuscript. NS and RH reviewed the final draft and edited English. All authors contributed to the article and approved the submitted version.

## Conflict of Interest

The authors declare that the research was conducted in the absence of any commercial or financial relationships that could be construed as a potential conflict of interest.

## Footnotes

1 https://www.eucast.org/

2 https://clsi.org/
